# Extravascular lung water assessed by transpulmonary single thermodilution and postmortem gravimetry in sheep

**DOI:** 10.1186/cc2974

**Published:** 2004-10-19

**Authors:** Mikhail Y Kirov, Vsevolod V Kuzkov, Vladimir N Kuklin, Kristine Waerhaug, Lars J Bjertnaes

**Affiliations:** 1 Research Fellow, Department of Anesthesiology, Faculty of Medicine, University of Tromsø, Tromsø, Norway; 2 Professor, Chairman of the Department of Anesthesiology, Faculty of Medicine, University of Tromsø, Tromsø, Norway

**Keywords:** acute lung injury, extravascular lung water, lipopolysaccharide, oleic acid, sheep

## Abstract

**Introduction:**

Acute lung injury is associated with accumulation of extravascular lung water (EVLW). The aim of the present study was to compare two methods for quantification of EVLW: transpulmonary single thermodilution (EVLW_ST_) and postmortem gravimetric (EVLW_G_).

**Methods:**

Eighteen instrumented and awake sheep were randomly assigned to one of three groups. All groups received Ringer's lactate (5 ml/kg per hour intravenously). To induce lung injury of different severities, sheep received *Escherichia coli *lipopolysaccharide 15 ng/kg per min intravenously for 6 hours (*n *= 7) or oleic acid 0.06 ml/kg intravenously over 30 min (*n *= 7). A third group (*n *= 4) was subjected to sham operation. Haemodynamic variables, including EVLW_ST_, were measured using a PiCCO*plus *monitor (Pulsion Medical Systems, Munich, Germany), and the last measurement of EVLW_ST _was compared with EVLW_G_.

**Results:**

At the end of experiment, values for EVLW_ST _(mean ± standard error) were 8.9 ± 0.6, 11.8 ± 1.0 and 18.2 ± 0.9 ml/kg in the sham-operated, lipopolysaccharide and oleic acid groups, respectively (*P *< 0.05). The corresponding values for EVLWI_G _were 6.2 ± 0.3, 7.1 ± 0.6 and 11.8 ± 0.7 ml/kg (*P *< 0.05). Ranges of EVLWI_ST _and EVLWI_G _values were 7.5–21.0 and 4.9–14.5 ml/kg. Regression analysis between *in vivo *EVLW_ST _and postmortem EVLW_G _yielded the following relation: EVLW_ST _= 1.30 × EVLW_G _+ 2.32 (*n *= 18, *r *= 0.85, *P *< 0.0001). The mean bias ± 2 standard deviations between EVLW_ST _and EVLW_G _was 4.9 ± 5.1 ml/kg (*P *< 0.001).

**Conclusion:**

In sheep, EVLW determined using transpulmonary single thermodilution correlates closely with gravimetric measurements over a wide range of changes. However, transpulmonary single thermodilution overestimates EVLW as compared with postmortem gravimetry.

## Introduction

Acute lung injury (ALI) of septic and non-septic origin is a frequent cause of mortality in critically ill patients. During ALI, the inflammatory process in the lungs may increase the microvascular pressure and permeability, resulting in an accumulation of extravascular lung water (EVLW) and development of pulmonary oedema [1]. However, it is difficult to estimate the amount of oedema fluid at the bedside. Clinical examination, chest radiography and blood gases have proven to be of limited value in quantifying pulmonary oedema [1-3]. Several techniques to assess EVLW have therefore been developed.

Among the various methods for measurement of EVLW, thermo-dye dilution has been used most frequently [4-8]. In animal models of lung oedema, this method has been evaluated by comparison with postmortem gravimetry, which is supposed to be the 'gold standard' of EVLW measurements [7-9]. In critically ill patients, fluid management guided by thermo-dye measured EVLW was associated with improved clinical outcome [10]. Hence, EVLW has been suggested to play a role as an independent predictor of the prognosis and course of illness [6,8,10]. However, the thermo-dye dilution method is relatively time consuming, cumbersome and expensive. For these reasons, the method has not gained general acceptance [4,5,7].

Use of a technique based on injection of a single thermo-indicator that can be detected using an indwelling arterial catheter was an appealing concept. Recent experimental and clinical studies have shown that EVLW assessed by single thermodilution (ST) exhibits good reproducibility and close agreement with the thermo-dye double indicator technique [11,12]. The ST method is simpler to apply, less invasive and more cost effective; all of these factors make it more suitable for use at the bedside. However, to date, this new method has been sparsely evaluated against gravimetry [13,14], and further validation is needed.

Thus, the aim of the present study was to evaluate the accuracy of the ST technique by comparing it with that of postmortem gravimetry (EVLW_G_) in conscious sheep, in which ALI was induced either by lipopolysaccharide (LPS) or by oleic acid (OA). Both of these models of ALI are reproducible and have been extensively described [7,9,11,15,16].

## Methods

### Surgical preparation and measurements

The study was approved by the Norwegian Experimental Animal Board and conducted in compliance with the European Convention on Animal Care. Eighteen yearling sheep weighing 27.5 ± 0.4 kg were instrumented, as a modification to previously described techniques [16-19], by inserting introducers into the left external jugular vein and common carotid artery. After 1–4 days of recovery, sheep were placed in an experimental pen. A thermodilution catheter (131HF7; Edwards Life Sciences, Irvine, CA, USA) was introduced into the pulmonary artery and a 4-Fr thermistor-tipped catheter (PV2014L16; Pulsion Medical Systems, Munich, Germany) into the carotid artery. The catheters were connected to pressure transducers (Transpac^®^III [Abbott, North Chicago, IL, USA] and PV8115 [Pulsion Medical Systems], respectively).

Mean pulmonary arterial pressure (PAP), pulmonary arterial occlusion pressure (PAOP) and right atrial pressure (RAP) were displayed on a 565A Patient Data Monitor (Kone, Espoo, Finland) and recorded on a Gould Polygraph (Gould Instruments, Cleveland, OH, USA). Heart rate, mean systemic arterial pressure, cardiac index (CI), systemic vascular resistance index, extravascular lung water index (EVLWI) assessed using the single thermodilution technique (EVLWI_ST_), pulmonary vascular permeability index (PVPI), global end-diastolic volume (GEDV) index (GEDVI), intrathoracic blood volume (ITBV) index (ITBVI) and blood temperature were determined at 1-hour intervals using a PiCCO*plus *monitor (Pulsion Medical Systems). Every value reported here is the mean of three consecutive measurements, each consisting of a 10 ml bolus of ice-cold 5% dextrose injected into the right atrium randomly during the respiratory cycle.

To estimate EVLW we used the following formula [12]: EVLW_ST _(ml) = ITTV - ITBV (where ITTV is the intrathoracic thermal volume). During clinical application of ST by means of the PiCCO monitor, ITBV is calculated as 1.25 × GEDV, the coefficient 1.25 being derived from critically ill patients [12]. However, in our previous investigations in sheep [17-19], in which ITBV was measured directly using the thermal-dye dilution technique, we found the coefficient to be 1.34 [14]. Thus, in the present study we used the corrected values of ITBVI, EVLWI_ST _and PVPI, based on the following equation: ITBVI = 1.34 × GEDVI.

Blood samples were drawn from the systemic arterial (a) and pulmonary arterial (v) lines and analyzed every two hours for blood gases and haemoglobin (Rapid 860; Chiron Diagnostics Corporation, East Walpole, MA, USA). The pulmonary vascular resistance index (PVRI), venous admixture (Qs/Qt), oxygen delivery index (DO_2_I) and oxygen consumption index were calculated as described previously [16,19,20].

### Experimental protocol

After establishing a stable baseline at time 0 hours, awake and spontaneously breathing sheep were randomly assigned to three experimental groups: a sham operated group (*n *= 4); a LPS group (*n *= 7), receiving an intravenous infusion of *Escherichia coli *O26:B6 LPS (Sigma Chemical, St. Louis, MO, USA) at 15 ng/kg per min for 6 hours; and an OA group (*n *= 7), in which sheep were subjected to an intravenous infusion of OA (Sigma Chemical) 0.06 ml/kg mixed with the animal's blood. The duration of the infusion of OA was 30 min.

During the experiment, all animals received a continuous infusion (5 ml/kg per hour) of Ringer's lactate, aiming to maintain intravascular volume at baseline levels. After the last measurements, at 2 hours in the OA group and at 6 hours in the sham-operated and the LPS groups, the sheep were anaesthetized and killed with a lethal dose of potassium chloride. Then, postmortem EVLWI (EVLWI_G_) was determined by gravimetry, as previously described [21-24].

### Statistical analysis

For each continuous variable, normality was checked using the Kholmogorov-Smirnov test. Data are expressed as mean ± standard error of the mean, and assessed by analysis of variance followed by Scheffe's test or test of contrasts, when appropriate. To evaluate the relationship between EVLWI_ST _and EVLWI_G_, we used linear regression and Bland-Altman analysis. *P *< 0.05 was considered statistically significant.

## Results

**Figure 1:**
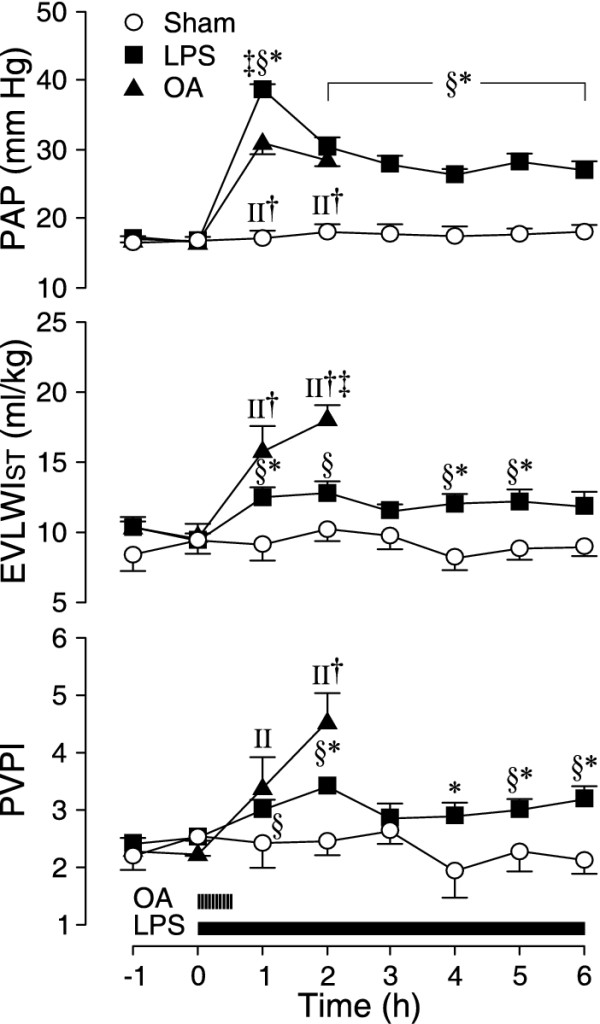
Changes in pulmonary haemodynamics and extravascular lung water in sheep. Data are expressed as mean ± standard error of the mean. *P < 0.05, LPS versus sham-operated group; ^†^*P *< 0.05, OA versus sham-operated group; ^‡^*P *< 0.05, LPS versus OA group; ^§^*P *< 0.05, versus t = 0 hours in LPS group; ^ll^*P *< 0.05 versus t = 0 hours in OA group. EVLWI_ST _= extravascular lung water index measured by single thermodilution; LPS = lipopolysaccharide; OA = oleic acid; PAP = pulmonary arterial pressure; PVPI = pulmonary vascular permeability index; Sham = sham-operated group.

**Figure 2:**
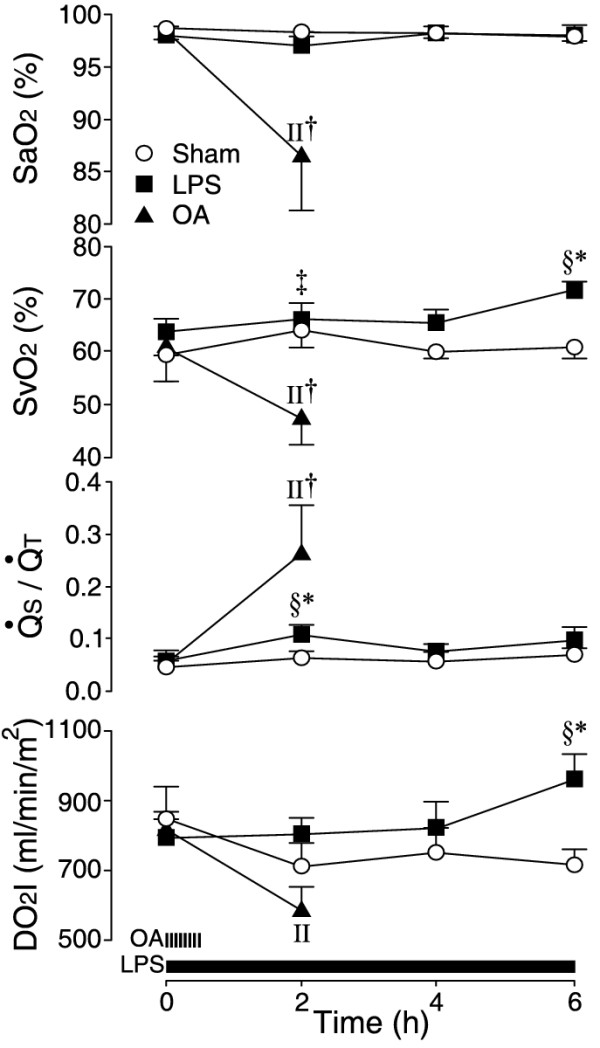
Changes in oxygenation variables in sheep. Data are expressed as mean ± standard error of the mean. *P < 0.05, LPS versus sham-operated group; ^†^*P *< 0.05, OA versus sham-operated group; ^‡^*P *< 0.05, LPS versus OA group; ^§^*P *< 0.05, versus t = 0 hours in LPS group; ^ll^*P *< 0.05, versus t = 0 hours in OA group. DO_2_I = oxygen delivery index; LPS = lipopolysaccharide; OA = oleic acid; Qs/Qt = venous admixture; SaO_2 _= arterial oxygen saturation; Sham = sham-operated; SvO_2 _= venous oxygen saturation.

All animals survived until the end of the experiments. At baseline no significant differences were found between groups, as shown in Figs 1 and 2, and Tables 1 and 2. In the sham-operated sheep, all variables remained unchanged throughout the study.

### Haemodynamic and extravascular lung water measurements

Figure 1 and Table 1 show that LPS and OA induced marked increments in PAP and PVRI, peaking at 1 hour and subsequently decreasing gradually to values significantly above the respective baselines and the corresponding values in the sham-operated group. PAOP and RAP also rose in both the LPS and the OA groups (*P *< 0.05; data not shown). In parallel, LPS increased EVLWI_ST _transiently by 20–35% (*P *< 0.05; Fig. 1). After OA administration, EVLWI_ST _rose to a maximum of 84% above baseline (*P *< 0.01). At the end of the experiment, EVLWI_ST _in the OA group had increased by 6.4 ml/kg and 9.3 ml/kg relative to the LPS and the sham-operated groups, corresponding to increments of 54% and 104%, respectively (*P *< 0.05). PVPI increased by 40% after LPS administration and by 90% after OA (*P *< 0.05; Fig. 1). GEDVI and ITBVI varied within 10–15% of baseline with no intergroup differences. As shown in Table 1, LPS caused tachycardia and a rise in CI accompanied by a slight increase in mean arterial pressure whereas systemic vascular resistance index decreased (*P *< 0.05). In contrast, in the OA group CI declined and systemic vascular resistance index increased relative to baseline (*P *< 0.05).

### Oxygenation and gas exchange

LPS caused significant increments in mixed venous oxygen saturation, DO_2_I and Qs/Qt (Fig. 2). OA decreased both arterial and venous oxygenation and reduced DO_2_I (*P *< 0.05). Oxygen consumption index did not change significantly (not shown). LPS caused a transient reduction in arterial carbon dioxide tension and a rise in pH (*P *< 0.05; Table 2). After OA, pH decreased (*P *= 0.04). The haemoglobin concentration as well as the body temperature rose only in the LPS group (*P *< 0.05).

### Linear regression and Bland-Altman analysis

**Figure 3:**
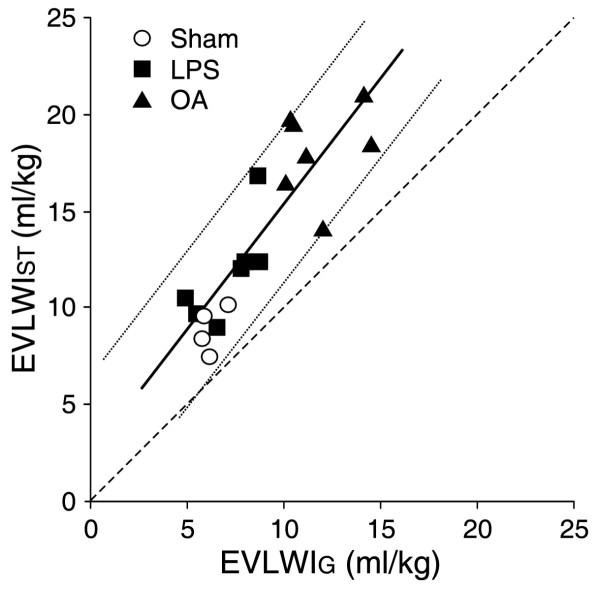
Linear regression analysis between extravascular lung water index (EVLWI) as determined by transpulmonary single thermodilution (EVLWI_ST_) and postmortem gravimetry (EVLWI_G_) in sheep. EVLWI_ST _= 1.30 × EVLW_G _+ 2.32 (*n *= 18, *r *= 0.85, *P *< 0.0001). Line of identity is dashed; 95% confidence intervals are indicated by solid lines. LPS, lipopolysaccharide; OA, oleic acid; Sham, sham-operated.

**Figure 4:**
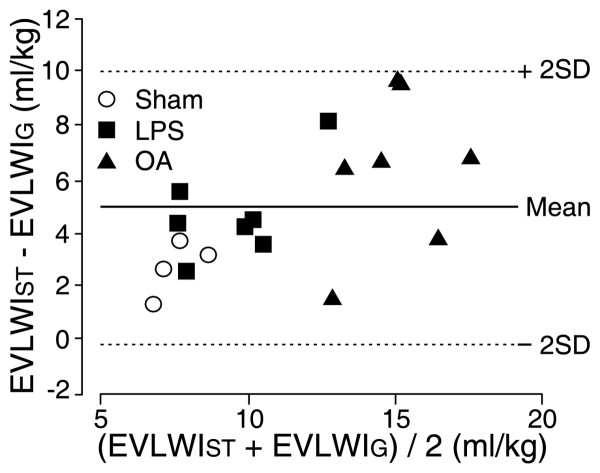
Bland-Altman plot for the extravascular lung water index (EVLWI) measured using transpulmonary single thermodilution (EVLWI_ST_) and postmortem gravimetry (EVLWI_G_) in sheep. The x-axis shows the mean of EVLWI measurements by single thermodilution and gravimetry. The y-axis shows the difference between the methods. The bold line indicates the value for the mean difference between EVLWI_ST _and EVLWI_G _(bias), and each dashed line indicates two standard deviations (SDs). Mean difference EVLWI_ST _- EVLWI_G _= 4.91 ml/kg (SD 2.54 ml/kg).

As shown in Fig. 3, the regression analysis between EVLW_ST _and postmortem EVLW_G _yielded the following relation: EVLWI_ST _= 1.30 × EVLW_G _+ 2.32 (*n *= 18, *r *= 0.85, *P *< 0.0001). Notably, the mean EVLWI_ST _at the end of experiment was higher than EVLWI_G_: 13.6 ± 1.1 ml/kg versus 8.7 ± 0.7 ml/kg (*P *= 0.0005). Ranges of EVLWI_ST _and EVLWI_G _values were 7.5–21.0 ml/kg and 4.9–14.5 ml/kg. According to the Bland-Altman analysis, the mean difference between EVLWI_ST _and EVLWI_G _was 4.91 ml/kg, with upper and lower limits of agreement (± 2 standard deviations) of +9.99 ml/kg and -0.17 ml/kg, respectively (Fig. 4). The difference between methods increased with increasing values of mean EVLWI (*n *= 18, *r *= 0.64; *P *= 0.005); the regression line equation was as follows: EVLWI_ST _- EVLWI_G _= 0.89 × ([EVLWI_ST _+ EVLWI_G_]/2) + 6.82.

### Postmortem gravimetry

**Figure 5:**
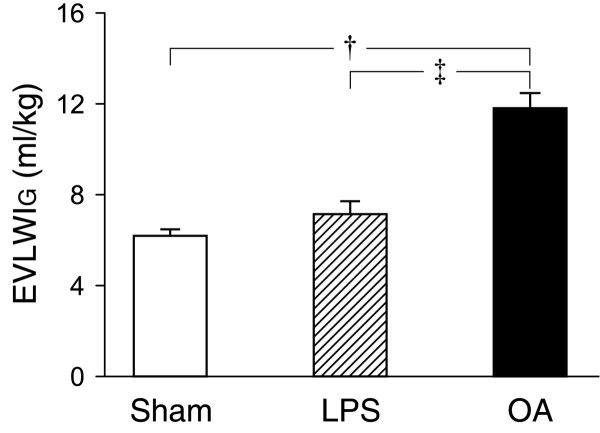
Gravimetric extravascular lung water index (EVLWI_G_) in sheep. Data are expressed as mean ± standard error of the mean. ^†^*P *< 0.05, OA versus sham-operated group; ^‡^*P *< 0.05, LPS versus OA group. LPS = lipopolysaccharide; OA = oleic acid; Sham = sham-operated group.

As shown in Fig. 5, EVLWI_G _in the OA group increased by 4.7 ml/kg and 5.6 ml/kg relative to the LPS and the sham-operated groups, amounting to increments by 65% and 90%, respectively (*P *= 0.001).

## Discussion

The present findings confirm that, in sheep, EVLW measured using the single transpulmonary thermodilution technique correlates closely with EVLW determined using postmortem gravimetry. However, EVLWI_ST _overestimates EVLWI_G_, with the degree of overestimation increasing with the severity of ALI.

A number of experimental and clinical studies focused on the potential role of EVLW as a guide to diagnosis and treatment of critically ill patients [3,6-14,25,26]. During pulmonary oedema, accumulation of EVLW occurs before any changes take place in blood gases, chest radiogram and, ultimately, pressure variables. In addition, the latter variables are nonspecific diagnostic tools that are influenced by a variety of factors [2,4,5,8]. Thus, Boussat and coworkers [3] recently demonstrated that, in sepsis induced ALI, commonly used filling pressures such as PAOP and RAP are poor indicators of pulmonary oedema. Rather than those measures, they recommended direct measurement of EVLW. Consistent with this, we found that EVLW, in contrast to RAP, correlates with markers of lung injury in human septic shock [26]. Victims of ALI, regardless of pathogenesis, have a significantly higher EVLW than do other patients [6,26]. Hence, measurement of EVLW supports the diagnosis and may even improve clinical outcomes when used cautiously in combination with treatment protocols that are known to hasten the resolution of pulmonary oedema [10,25].

Instrumented awake sheep represents a stable experimental model for measuring cardiopulmonary variables, as demonstrated in the sham-operated group in the present study as well as by other investigators [15,27]. The model can be used to assess different interventions during ALI.

Consistent with previous investigators [15,17,27], we observed that infusion of LPS and OA caused pulmonary hypertension, increased EVLW and impaired gas exchange. Despite increments in PAP, PAOP and PVRI, both ITBV and GEDV remained constant whereas PVPI (an index of microvascular permeability, calculated as the ratio of EVLW to pulmonary blood volume) increased significantly. Thus, the haemodynamic responses to LPS and OA are not purely hydrostatic but may also manifest as noncardiogenic permeability pulmonary oedema [13,15-18,27,28].

In the present study lung oedema was significantly more severe in the OA group than in the LPS group, which is consistent with the findings of other investigators [29]. In fact, OA causes acute haemorrhagic alveolitis, which may lead to acute endothelial and alveolar necrosis and a severe proteinaceous oedema [30]. In contrast, the LPS-induced ALI is initiated by accumulation of granulocytes and lymphocytes in the pulmonary microcirculation that results in more moderate damage to endothelial cells and lung oedema [31].

Lung injury in the LPS group was accompanied by a hyperdynamic circulatory state, which was manifested by systemic vasodilation and increments in CI and DO_2_I toward the end of the experiment. In contrast, in the OA group we observed cardiac depression and systemic vasoconstriction. This is consistent with previous investigations of LPS and OA [18,27,30,32]. Thus, ovine models exhibit a scatter of cardiopulmonary changes from normal in the sham-operated group to mild or moderate ALI in endotoxaemic sheep and moderate to severe ALI in animals subjected to OA.

The significant correlation of EVLWI_ST _and EVLWI_G _observed in the present study is consistent with findings of Katzenelson and coworkers [13], who validated EVLWI_ST _versus postmortem gravimetry in dogs [13]. However, those investigators did not specifically assess the relationship between EVLWI_ST _and EVLWI_G _in sepsis-induced ALI. In addition, their study was performed in anaesthetized and mechanically ventilated animals; hence, further investigation of the correlation in a conscious state was required. Recently, ST has been evaluated against the thermo-dye dilution method in both experimental and clinical settings [11,12]. The studies revealed a close agreement between the techniques. Thus, we believe that injection of cold saline can provide valuable information about the EVLW content and the severity of pulmonary oedema.

During ALI, both ST and postmortem gravimetry demonstrated similar relative increases in EVLWI as compared with sham-operated animals. However, we noticed that ST overestimates the absolute values of EVLWI compared with the gravimetric technique – a discrepancy that increased with progression of pulmonary oedema. This finding could be accounted for by heat exchange of the thermal indicator with extravascular intrathoracic structures, such as the walls of the large vessels and the myocardium, and by recirculation of the indicator [8]. In addition, the coefficients for calculation of EVLWI_ST _and ITBV may vary with weight and age, as well as between animal species [11]. Consequently, in the experimental setting EVLWI_ST _requires a specific correction. In the present study we replaced the coefficient 1.25 used in humans in the ITBVI equation (i.e. ITBVI = 1.25 × GEDVI) with the recalculated 'ovine' coefficient 1.34 [14], which is based on 426 measurements in 48 animals [17-19].

In contrast to ST, the thermo-dye dilution technique runs the risk of underestimating EVLW in comparison with gravimetry [4]. This underestimation increases during ALI caused by instillation of hydrochloric acid into the airways, and has been explained by redistribution of pulmonary blood flow away from the oedematous areas. The redistribution is thought to prevent indicator diffusion and consequently to prevent detection of oedema [7]. In addition, detection of EVLW by thermo-dye dilution can be impaired by changes in CI as well as by positive end-expiratory pressure during mechanical ventilation [8,28].

Compared with other techniques for assessment of EVLW, ST may underestimate EVLW during pulmonary oedema due to intratracheal instillation of saline, although it is an accurate method in normal lungs [33]. However, intratracheal instillation of saline can also be criticized because a proportion of the fluid is rapidly absorbed and obscured from detection [34].

Notably, the use of postmortem gravimetry as the reference method for evaluating pulmonary oedema also has limitations [21,33]. For example, the method only allows one measurement and is therefore of no use in following variations over time. The application of gravimetry is limited almost exclusively to experimental studies. The comparison of gravimetric measurement with results of other techniques for determination of EVLW can be influenced by the duration from death to removal of the lungs and by pathophysiological changes in the lungs after cardiac arrest. Thus, the gravimetric technique can underestimate the real value of EVLWI because of partial reabsorption of fluid before excision of the lungs.

## Conclusion

The determination of EVLW by ST in sheep correlates closely with gravimetric measurements over a wide range of changes, and thus it may potentially be of benefit in quantifying lung oedema in critically ill patients. However, compared with postmortem gravimetry, single transpulmonary thermodilution overestimates the absolute values of EVLW. Thus, further studies are warranted to evaluate the accuracy of this method for managing ALI in humans.

## Key messages

• 	In sheep, extravascular lung water assessed by transpulmonary single thermodilution correlates closely with gravimetric measurements over a wide range of changes.

• 	Despite a moderate overestimation of the extravascular lung water content compared with post-mortem gravimetry, single thermodilution can be a useful tool for assessment of pulmonary oedema during ALI.

## Competing interests

This study was supported by Helse Nord (Norway), project number 4001.721.132; departmental funds, the Department of Anesthesiology, University Hospital of North Norway; and Pulsion Medical Systems (Germany).

## Author contributions

MYK participated in the design of study, performed statistical analysis, and drafted the manuscript. VVK participated in the design of study, performed statistical analysis, and prepared the figures. VVK and KW participated in the design of study. LJB participated in the design of study and provided coordination. All authors read and approved the final manuscript.

## Abbreviations

ALI = acute lung injury; CI = cardiac index; DO_2_I = oxygen delivery index; EVLW = extravascular lung water; EVLWI = extravascular lung water index; GEDV = global end-diastolic volume; GEDVI = global end-diastolic volume index; ITBV = intrathoracic blood volume; ITBVI = intrathoracic blood volume index; LPS = lipopolysaccharide; OA = oleic acid; PAOP = pulmonary arterial occlusion pressure; PAP = pulmonary arterial pressure; PVPI = pulmonary vascular permeability index; PVRI = pulmonary vascular resistance index; Qs/Qt = venous admixture; RAP = right atrial pressure; ST = single thermodilution.

## Figures and Tables

**Table 1:** Haemodynamics during acute lung injury in sheep

Parameter	Group	Time point (hours)						
		
		0	1	2	3	4	5	6
PVRI (dyne·s/cm^5 ^per m^2^)	Sham	117 ± 14	131 ± 19	141 ± 10	145 ± 11	114 ± 21	133 ± 13	151 ± 16
	LPS	115 ± 6	284 ± 20*^†^	240 ± 21*^†^	198 ± 31^†^	193 ± 26*^†^	199 ± 23*^†^	182 ± 16*^†^
	OA	103 ± 9	351 ± 64^‡§^	300 ± 45^‡§^	-	-	-	-
GEDVI (ml/m^2^)	Sham	570 ± 46	601 ± 68	572 ± 43	566 ± 12	661 ± 74	607 ± 67	655 ± 60
	LPS	571 ± 23	620 ± 57	564 ± 32	579 ± 42	598 ± 38	624 ± 42	615 ± 37
	OA	646 ± 38	629 ± 60	590 ± 55	-	-	-	-
ITBVI (ml/m^2^)	Sham	764 ± 62	806 ± 91	766 ± 58	759 ± 16	886 ± 99	813 ± 90	878 ± 81
	LPS	765 ± 30	831 ± 76	756 ± 43	776 ± 57	801 ± 51	836 ± 57	825 ± 49
	OA	866 ± 51	912 ± 42	790 ± 74	-	-	-	-
HR (beats/min)	Sham	106 ± 6	104 ± 8	96 ± 7	91 ± 5	99 ± 6	98 ± 11	97 ± 5
	LPS	96 ± 4	122 ± 6^†^	109 ± 6	109 ± 4*^†^	109 ± 8	122 ± 4*^†^	130 ± 5*^†^
	OA	111 ± 5	104 ± 13	102 ± 13	-	-	-	-
CI (l/min per m^2^)	Sham	5.7 ± 0.3	5.5 ± 0.3	5.2 ± 0.3	5.1 ± 0.2	5.4 ± 0.4	5.2 ± 0.3	5.3 ± 0.3
	LPS	5.7 ± 0.1	7.3 ± 0.5*^†ll^	5.9 ± 0.2	5.8 ± 0.3	5.6 ± 0.4	6.2 ± 0.3*	6.8 ± 0.2*^†^
	OA	6.1 ± 0.3	4.5 ± 0.3^§^	4.6 ± 0.5	-	-	-	-
MAP (mmHg)	Sham	102 ± 5	101 ± 6	101 ± 6	101 ± 5	102 ± 5	102 ± 5	101 ± 4
	LPS	94 ± 4	104 ± 5^†^	105 ± 3^†^	106 ± 4^†^	105 ± 3^†^	104 ± 5	100 ± 6
	OA	94 ± 4	101 ± 2	104 ± 4^§^	-	-	-	-
SVRI (dyne·s/cm^5 ^per m^2^)	Sham	1453 ± 101	1496 ± 125	1589 ± 138	1536 ± 105	1579 ± 109	1607 ± 119	1681 ± 169
	LPS	1410 ± 81	1128 ± 102^†^	1352 ± 62	1515 ± 107	1428 ± 101	1308 ± 79	1126 ± 58*^†^
	OA	1266 ± 63	1847 ± 368	1670 ± 147^§^	-	-	-	-

Data are expressed as mean ± standard error of the mean. *P < 0.05, LPS versus sham-operated group; ^†^*P *< 0.05, versus t = 0 hours in LPS group; ^‡^*P *< 0.05, OA versus sham-operated group; ^§^*P *< 0.05, versus t = 0 hours in OA group; ^ll^*P *< 0.05, LPS versus OA group. CI, cardiac index; GEDVI, global end-diastolic volume index; HR, heart rate; ITBVI, intrathoracic blood volume index; LPS, lipopolysaccharide; MAP, mean arterial pressure; OA, oleic acid; PVRI, pulmonary vascular resistance index; Sham, sham-operated; SVRI, systemic vascular resistance index.

**Table 2:** Gas exchange during acute lung injury in sheep

Parameter	Group	Time point (hours)			
		
		0	2	4	6
pHa	Sham	7.52 ± 0.03	7.53 ± 0.02	7.50 ± 0.02	7.50 ± 0.02
	LPS	7.48 ± 0.01	7.50 ± 0.02	7.55 ± 0.02*	7.53 ± 0.02
	OA	7.50 ± 0.01	7.44 ± 0.03^†^	-	-
PaCO_2 _(mmHg)	Sham	38.7 ± 2.7	36.4 ± 2.1	36.4 ± 1.6	37.4 ± 1.0
	LPS	39.7 ± 1.4	38.8 ± 1.5	32.6 ± 0.6*^‡^	33.1 ± 1.0*^‡^
	OA	36.4 ± 1.1	42.5 ± 3.7	-	-
Haemoglobin (g/dl)	Sham	10.7 ± 0.9	10.1 ± 0.6	10.2 ± 0.5	10.3 ± 0.5
	LPS	10.4 ± 0.6	10.4 ± 0.6	11.0 ± 0.7*	10.9 ± 0.6
	OA	10.3 ± 0.4	10.7 ± 0.5	-	-
Blood temperature (°C)	Sham	39.3 ± 0.1	39.2 ± 0.1	39.3 ± 0.1	39.3 ± 0.1
	LPS	39.3 ± 0.1	40.0 ± 0.1*^‡^	41.3 ± 0.1*^‡^	41.0 ± 0.1*^‡^
	OA	39.5 ± 0.1	39.6 ± 0.1	-	-

Data are expressed as means ± standard error of the mean. *P < 0.05, versus t = 0 hours in LPS group; ^†^*P *< 0.05, OA versus sham operated group; ^‡^*P *< 0.05, LPS versus sham-operated group. LPS, lipopolysaccharide; OA, oleic acid; PaCO_2_, arterial carbon dioxide tension; Sham, sham-operated.
